# Effects of Three Artificial Diets on Life History Parameters of the Ladybird Beetle *Stethorus* *gilvifrons*, a Predator of Tetranychid Mites

**DOI:** 10.3390/insects11090579

**Published:** 2020-09-01

**Authors:** Jafar Ebrahimifar, Parviz Shishehbor, Arash Rasekh, Seyed Ali Hemmati, Eric W. Riddick

**Affiliations:** 1 Department of Plant Protection, Faculty of Agriculture, Shahid Chamran University of Ahvaz, Ahvaz 61357-43311, Iran; jafar.ebrahimi@alumni.ut.ac.ir (J.E.); pshishehbor@scu.ac.ir (P.S.); a.rasekh@scu.ac.ir (A.R.); 2 Agricultural Research Service, USDA, Stoneville, MS 38776, USA; eric.riddick@usda.gov

**Keywords:** biological control, *Stethorus gilvifrons*, coccinellids, life table, mass production, tetranychid mites, 2,4-dihydroxybenzoic acid

## Abstract

**Simple Summary:**

The ladybird beetle *Stethorus gilvifrons* is an important natural enemy of spider mites, which are pests of numerous agricultural crops. Mass production of *S*. *gilvifrons* would be necessary to generate large numbers of individuals for augmentative biological control. Using alternative prey/foods or artificial diets as a substitute to natural prey (spider mites) would be important in the cost-effective production of *S*. *gilvifrons*. The objectives of the current study were to determine the developmental and reproductive characteristics of *S. gilvifrons* fed on different artificial diets. Artificial diets consisted of sucrose, honey, royal jelly, agar, yeast, date palm pollen supplemented with hen’s egg yolk (AD1, as basic diet), *Ephestia kuehniella* eggs (AD2), or *E*. *kuehniella* eggs and 2,4-dihydroxybenzoic acid (AD3). Adults and larvae of *S.* *gilvifrons* performed best on AD3, indicating the potential of this artificial diet for mass rearing of this important predatory ladybird beetle.

**Abstract:**

Background: The ladybird beetle *Stethorus gilvifrons* (Mulsant) is an important natural enemy of tetranychid mites and functions as a biological control of these plant pests. The development, survival and reproduction of *S. gilvifrons* were studied when fed on three artificial diets. The components of the artificial diet that *S. gilvifrons* could be reared successfully on for one generation with no use of tetranychid mites were examined. Methods: Artificial diets consisted of sucrose, honey, royal jelly, agar, yeast, date palm pollen supplemented in different diets with hen’s egg yolk (AD1, as basic diet), *Ephestia kuehniella* Zeller eggs (AD2), or *E*. *kuehniella* eggs and 2,4-dihydroxybenzoic acid (AD3). Results: Adults and larvae of *Stethorus* *gilvifrons* fed on AD1 had a shorter immature development and preoviposition periods than those fed on AD2 and AD3. The total number of deposited eggs was significantly higher for the females fed on AD3 than on the other diets. The intrinsic rate of increase (*r*) of *S. gilvifrons* was highest on AD3, followed by AD2, and AD1. Conclusion: *Stethorus gilvifrons* performed best on AD3, indicating the potential of this artificial diet for the mass rearing of this important predatory ladybird beetle.

## 1. Introduction

The strawberry spider mite, *Tetranychus turkestani* Ugarov and Nikolskii (Acarina: Tetranychidae) is an important pest of Solanaceae, Cucurbitaceae and Fabaceae plants in Khuzestan province, southwestern Iran [1,2]. *T. turkestani* is often found on field bindweed (*Convolvulus arvensis* L.) in field margins, and on several other weeds, which may serve as reservoirs. The strawberry spider mite has multiple generations during the growing season, tolerates high temperatures and low humidity, and has a short generation time of approximately 6–7 days [3]. Different developmental stages of *T. turkestani* initially feed on the lower leaf surface but can cover an entire plant as populations increase. Early damage symptoms are the appearance of chlorotic stipples on the leaves, but large areas will turn yellow as feeding damage builds up. Leaves also may become bronzed and the plant can defoliate. Webbing is often very evident, giving a bright appearance to the plant [4].

An effective strategy to manage *T*. *turkestani* may involve the deployment of natural enemies, such as ladybird beetles (Coleoptera: Coccinellidae). The coccinellid tribe Stethorini includes the genera *Stethorus* and *Parastethorus* and consists of approximately 90 described species that are known as specialist mite predators. These ladybird beetles feed on spider mites (Tetranychidae) and false spider mites (Tenuipalpidae) [4,5,6,7]. As specialist predators, *Stethorus* spp. can decrease the density of tetranychid mite populations, as shown for *Stethorus punctillum* Weise on vineyards in Europe [8,9], citrus in China [10], and raspberries in Canada [11,12]. Other species such as *Stethorus punctum punctum* (LeConte) on apple in the USA [13], *Stethorus punctum picipes* Casey on avocado in the USA [14,15], and *Stethorus gilvifrons* (Mulsant) on tea in India [16] and date palm in Iran [17] are also known to reduce tetranychid mite density.

*Stethorus gilvifrons* was reported as a native predator of different spider mites from Iran [1] and other countries in western Asia [6,18,19]. It occurs commonly in fields of sugarcane [20], date palm [21,22], and castor bean [23]. It is known as a suitable candidate for the biological control of several tetranychid mite species [6,7,19]. It has high host-finding ability, high dispersal potential, voracious appetite and the adults are long lived [20,24]. Although different active stages of *S. gilvifrons* feed on different developmental stages of tetranychid mites by sucking their body fluid, they prefer the early developmental stages and especially the egg stage [6,25,26].

A review of the literature indicates that the augmentative release of natural enemies is an effective strategy for pest management, particularly in protected plant cultivation, i.e., glasshouses or greenhouses [27,28,29]. Mass production is essential in order to use entomophagous insects, including predatory ladybird beetles in augmentative biological control, since large numbers of individuals are required for effective biological control [30,31]. Old-fashioned rearing procedures are dependent on natural prey and involve a great number of preys along with their host plants. Supporting the progress of such a multifaceted tritrophic rearing scheme encompasses great challenges [32,33,34]. In view of the necessity for cost reduction, using factitious prey/foods or artificial diets as a substitute to natural prey is an important measure in the production of predatory lady beetles [34,35,36].

Little is known about rearing *Stethorus* spp. on alternative foods. In an early study, Smirnoff [36] reported that *S. punctum* and 17 other coccinellid beetles were successfully reared in the laboratory on a diet containing cane sugar, honey, agar and royal jelly plus their natural prey in powder form. Wheat germ diet plus honey was used by Colburn [37] for rearing *S. punctum*. This diet significantly improved adult survival over two weeks compared to sugar water alone. *S*. *punctum* was not able to reproduce on either of the two diets mentioned above. Ebrahimifar et al. [38] revealed that *S. gilvifrons* can be successfully reproduced and reared on *Ephestia kuehniella* Zeller eggs plus plant pollens (date palm, maize, and bee pollens) as alternative foods. Likewise, they found the combination of *E. kuehniella* eggs plus date palm pollen holds promise as an alternative food for the mass production of *S. gilvifrons*. Moreover, the authors discovered that date palm pollen was scattered in Ahvaz city and readily available as a food source for *S. gilvifrons* adults [38].

In the previous study by Ebrahimifar et al. [38], factitious diets were only tested as food for *S*. *gilvifrons*. The objectives of the current study were to determine the developmental and reproductive characteristics of *S. gilvifrons* fed on different artificial diets. The hypothesis that *S. gilvifrons* can develop and reproduce on artificial diets devoid of tetranychid mites was tested in laboratory bioassays. Also, previous research showed that the phenolic compound 2,4-dihydroxybenzoic acid (DHBA) was a weak oviposition stimulant for the aphidophagous ladybird beetle *Coleomegilla maculata* (DeGeer) [39]. Consequently, the hypothesis that DHBA could increase reproduction (oviposition) in *S*. *gilvifrons* was also tested in this study. This information could facilitate the mass rearing of *S. gilvifrons* for the augmentative biological control of tetranychid mites on crop plants in Iran and elsewhere.

## 2. Materials and Methods

### 2.1. Stock Colony of T. turkestani and S. gilvifrons

*Tetranychus turkestani* used in this study originated from a weed (*C. arvensis*) at the Faculty of Agriculture, Shahid Chamran University of Ahvaz, Ahvaz, Iran. A stock colony of *T. turkestani* was maintained on the seedlings of cowpea (*Vigna unguiculata* (L.) Walp.) grown from seeds and transplanted into compost in plastic pots (20 cm diam.). Infested plants were held in wooden-framed rearing cages (120 × 60 × 60 cm) covered with nylon mesh of 210 µm aperture. The cages were maintained in a laboratory at 25 ± 2 °C, 50 ± 5% RH and a 14:10 (L:D) h with illumination (4000 lux) provided by fluorescent lamps. New plants were introduced as required.

Adult *S. gilvifrons* females and males used in this study were originally collected from a sugarcane field around Ahvaz in July 2018 and cultured in conditions identical to *T. turkestani*, using cowpea plants infested with different developmental stages of *T. turkestani.* Every week new cowpea plants infested with *T. turkestani* were added to the cages. These cages were also kept in the laboratory at similar conditions as above. The identity of *T. turkestani* and *S. gilvifrons* were confirmed by K. Kamali and H. Hodek, respectively, and voucher specimens were deposited in the insect collection of Shahid Chamran University of Ahvaz, Ahvaz, Iran.

### 2.2. Artificial Diets and Their Compositions

The artificial diet ingredients used in this study were modified from an original diet developed by Smirnoff [36]. We prepared the artificial diets (AD) of three different compositions. The ingredients of each artificial diet (AD1, AD2 and AD3) were presented separately in Table 1. Honey (Natural honey, Kouhrang Shop, Ahvaz, Iran), sucrose, and agar (CAS.QB-42-0223, Sigma Aldrich Co., St. Louis, MO, USA) were dissolved into the distilled water, after which the yeast extract (Top Levure, Sigma Aldrich Co., St. Louis, MO, USA) and hen’s egg yolk were added. All ingredients were then blended using a magnetic stirrer (RH basic 2, IKA, Staufen, Germany). Date palm pollen and date palm pollen plus *E. kuehniella* eggs were prepared by finely grinding 1.5 g date palm pollen (AD1) and 1.5 g date palm pollen plus 1.5 g *E. kuehniella* eggs (AD2 and AD3) to dust in a ceramic mortar. Then, the resulting mixture was added to the basic diet and mixed again. The final mixture was transferred to small plastic tubes and stored in a freezer at −18 °C. For AD1, AD2, and AD3, the fresh diet was prepared every week and kept in a refrigerator at 5 °C. *Ephestia kuehniella* eggs used in this study were purchased from a colony at Golestan Mooud Insectary Company, Ahvaz, Iran and reared continuously according to the method described by Brindley [40]. Eggs were collected daily and stored in a refrigerator at 4 °C for less than two weeks before use. Date palm pollen was obtained from a rural supplier in Ahvaz city, Iran. The pollen was kept in a freezer at −18 °C before use. DHBA (97% pure powder, product no. D109401-5G) was purchased from Sigma-Aldrich Co. (St. Louis, MO, USA).

### 2.3. Experimental Setup

Using a completely randomized design, 54, 58, and 60 newly hatched (<12 h) larvae of *S. gilvifrons* were transferred to Petri dish arenas (one larva per Petri dish) (60 mm × 16 mm) and 0.3 g from one of the three food diets AD1, AD2, and AD3, respectively. All diets were supplied ad libitum and refreshed every two days. To obtain data on the duration of the immature development of *S. gilvifrons* and the survival rate, observations were made every 24 h until all individuals had reached adulthood. The developmental stage of each individual was determined based on the presence of exuviae in the Petri dish. Upon adult emergence, the adults were sexed and weighed using an N-202 precise digital balance (AND Company, Tokyo, Japan) with a precision of 0.001 mg. Females and males reared from the same larval food regime were paired and supplied with the same diets as in the larval stages in individual Petri dishes (n = 20 for each treatment). A metal net wadding was as an oviposition substrate in the base of each Petri dish. Males that died during the experiment were replaced with males that had been maintained on the same diet. Adults were observed daily to determine the preoviposition and oviposition period, longevity and fecundity. Progeny from females of the same age were transferred to new Petri dishes and fed with the same diet as their parents to determine the offspring sex ratio. Adults that escaped or died as a result of manipulation were excluded from data analysis. The experiments were conducted in a growth chamber at 30 ± 1 °C, 65 ± 5% RH and a 16:8 (L:D) photoperiod. *T. turkestani* and *S. gilvifrons* were mostly active during late spring and summer seasons in Khuzestan province, southwestern Iran. During this period, the weather was very hot and dry and the mean ambient temperature fluctuated around 30 °C. Since both *T. turkestani* and *S. gilvifrons* were adapted to high temperature (30 °C), this temperature was used for the experiment.

### 2.4. Statistical Analysis

The datasets were first verified for normal distribution by the Kolmogorov–Smirnov test. The developmental time, immature survival, adult body weight, preoviposition period, oviposition period, postoviposition period, adult longevity, total fecundity and egg hatchability of *S. gilvifrons* reared on different diets were analyzed using a one-way analysis of variance (one-way ANOVA), after the arcsine transformation of an immature survival rate and egg hatch rate data. Means were separated using Tukey’s HSD test at a *p* < 0.05 significance level [41]. A Student’s *t*-test was used to analyze the differences in sex ratio (50% female) of *S. gilvifrons* fed on different diets.

An age-stage, two-sex life-table procedure [42] was selected for the data analysis to account for the variable developmental rates among the individuals and the stages of development of the *S. gilvifrons* reared on different artificial diets. Population growth parameters including net reproductive rate (*R_o_*), gross reproductive rate (*GRR*), intrinsic rate of natural increase (*r*), finite rate of increase (*λ*) and mean generation time (*T*) were calculated with the TWOSEX-MSChart program [43]. Standard errors of the population growth parameters were obtained using the bootstrap technique and multiple comparisons were made by the paired bootstrap test with 100,000 samples.

## 3. Results

*Stethorus gilvifrons* larvae and pupae completed their development on all the diets tested. Developmental times of both *S. gilvifrons* males and females were not significantly affected by diet (males, F = 0.97; df = 2, 73; *p* = 0.39; females, F = 0.94; df = 2, 73; *p* = 0.41; Table 2). Immature survival, from the egg stage to the adult stage, of *S. gilvifrons,* did not differ among the diets and ranged from 64.33 to 70.23% (F = 0.46; df = 2, 57; *p* = 0.65). The body weight of males was not significantly affected by larval diet (F = 1.41; df = 2, 42; *p* = 0.25), however, the body weight of females indicated significant differences (F = 4.18; df = 2, 42; *p* ≤ 0.05).

The diet significantly affected the duration of the preoviposition period (F = 3.45; df = 2, 31; *p* ≤ 0.05). Females fed on AD1 and AD2 had significantly shorter preoviposition periods than those fed on AD3 (Table 3). However, the oviposition period and longevity were significantly longer on the AD3 than on AD1 diet (oviposition period: F = 3.59; df = 2, 31; *p* ≤ 0.05; Table 3 and female longevity: F = 1.02; df = 2, 31; *p* ≤ 0.05; Table 3). Moreover, adult males and females fed on AD1 lived for fewer days than those fed on AD2 and AD3. In addition, females had a longer preoviposition period. Total fecundity was significantly higher for the females offered AD3 versus the other diets (F = 11.01; df = 2, 31; *p* ≤ 0.001; Table 3). The diet had no influence on the egg hatch (ranging 66–72%; Table 3) (F = 1.07; df = 2, 131; *p* = 0.39) or the sex ratio of offspring (ranging 45–48% female; Table 3) (F = 0.28; df = 2, 88; *p* = 0.76).

Differences in the developmental and reproductive traits were reflected in life table statistics. The intrinsic rate of increase (*r*) in *S. gilvifrons* was highest on AD3, followed by AD2, and lowest on AD1 (Table 4). Similarly, the generation time (*T*) was shortest for the females fed AD3 rather than the other diets.

## 4. Discussion

The availability of the cheap factitious and artificial diets can strongly help in making the mass rearing of beneficial natural enemies more cost-effective. In the present study, we used a mix of different foods such as date palm pollen, factitious prey *E. kuehniella* eggs, honey, yeast extract, royal jelly, agar and 2,4-dihydroxybenzoic acid to maintain the predatory ladybird, *S. gilvifrons*. Promising results with regards to the survival and reproductive performance showed that these artificial diets have good potential to be used in the mass rearing of the predator. The rearing *S. gilvifrons* on these artificial diets could be less time-consuming, less labor intensive, and more cost-effective than using tetranychid mites as the sole food source.

No data were available in the literature, to our knowledge, on immature developmental time, longevity, fecundity or the intrinsic rate of the increase in *Stethorus* spp. feeding on artificial diets. Ebrahimifar et al. [38] studied the developmental time, survival and reproductive performance of *S. gilvifrons* fed on different factitious diets and reported that a mixture of date palm pollen plus *E. kuehniella* eggs was the best diet, among the different factitious diets tested, for *S. gilvifrons* development and reproduction. The results of the current study indicated that *S. gilvifrons* fed on AD1 that lacked *E. kuehniella* eggs had very close female developmental time (15.50 days), immature survival (70.23%), female longevity (31.18 days), total fecundity (42.09 eggs), egg hatch (72.0%), sex ratio (53.62% female) and *r* (0.119 d^−1^) values compared to those reported by Ebrahimifar et al. [38], suggesting that the nutritional quality of two the artificial diets (AD1 and AD2) was similar. On the other hand, the *r* values of *S. gilvifrons* on the artificial diet (AD1) calculated here (0.119 d^−1^) or on a factitious diet (date palm pollen plus *E. kuehniella* eggs) reported by Ebrahimifar et al. [38] (0.136 d^−1^) were lower than the values reported by other workers for this predator on its natural prey: 0.171 d^−1^ on *T. turkestani* [26], 0.191 d^−1^ on *Tetranychus urticae* Koch [44], 0.189 d^−1^ on *Oligonychus afrasiaticus* (McGregor) [21], and 0.152 d^−1^ on *Tetranychus cinnabarinus* (Boisduval) [18].

The results of our experiments showed that the number of larval instars of *S. gilvifrons* fed on artificial diets were the same as those reported for this species fed natural prey *Oligonychus coffeae* (Nietner) [16], *T. turkestani* and *Eutetranychus orientalis* Klein [26], *T. urticae* [44], *O. afrasiaticus* [21], and *T. cinnabarinus* [18]. It has been noted that inadequate foods can increase the number of larval instars [45,46], therefore the artificial diets presented to *S. gilvifrons* in the current study had adequate nutritional quality in comparison to the natural prey (tetranychid mites).

According to our findings, DHBA (a weak oviposition stimulant) increased progeny production in *S. gilvifrons*. Several phenolic compounds including DHBA, quercetin, taxifolin, and naringenin have stimulated oviposition behavior in the aphidophagous coccinellid, *C. maculata*, in the laboratory [40,47,48].

## 5. Conclusions

In conclusion, all the artificial diets tested, particularly AD3, supported the development, survival and reproduction of *S. gilvifrons* in the absence of tetranychid mites indicating the potential of artificial diets to mass rear this economically important biological control agent. However, nutritional imbalances within the diets could be expressed in subsequent generations [49]. Therefore, the development and reproduction of *S. gilvifrons* fed on AD3 should be evaluated over subsequent generations. Furthermore, *E. kuehniella* eggs were very expensive (around 400 EUR/kg) [50], which hampered the cost effectiveness of AD3. Rearing *S. gilvifrons* on artificial diets could be less labor intensive and more cost effective than using tetranychid mites as the sole food source. Thus, future work is necessary to reduce the percentage of *E. kuehniella* eggs in the diet or to replace it with a less expensive food source.

## Figures and Tables

**Table 1:** Ingredients of three artificial diets supplied to *S. gilvifrons.*

Ingredient	Artificial Diets (AD)		
	AD1	AD2	AD3
Sterilized distilled water	50 mL	50 mL	50 mL
Agar	1.3 g	1.3 g	1.3 g
Sugar	10 g	10 g	10 g
Royal jelly	1.5 g	1.5 g	1.5 g
Honey	4.5 g	2.5 g	4.5 g
Date palm pollen	1.5 g	1.5 g	1.5 g
Hen’s egg yolk	2.5 g	-	-
Yeast	-	1 g	1 g
*E*. *kuehniella* egg	-	1.5 g	1.5 g
2,4-dihydroxybenzoic acid	-	-	0.3 g

**Table 2:** Mean ± SE immature survival, developmental time, and adult body weight of *S. gilvifrons* fed on three different artificial diets.

Diet	Immature Survival (%) (n = 20)	Developmental Time (Days) (n = 25)		Body Weight (mg) (n = 15)	
		Male	Female	Male	Female
AD1	64.33 ± 4.13 a	14.07 ± 1.88 b	14.86 ± 1.86 a	0.123 ± 0.007 a	0.132 ± 0.009 b
AD2	67.01 ± 2.28 a	13.86 ± 1.46 b	14.60 ± 0.97 a	0.144 ± 0.011 a	0.158 ± 0.012 ab
AD3	70.23 ± 4.23 a	15.13 ± 1.88 a	15.50 ± 1.32 a	0.147 ± 0.014 a	0.180 ± 0.015 a

Means followed by a different letter in a column are significantly different (Tukey HSD test at *p* ≤ 0.05).

**Table 3:** Mean ± SE adult longevity, female periods, fecundity, egg hatching, and the sex ratio of *S. gilvifrons* fed on three different artificial diets.

Diet	Male Longevity (Days)	Female Longevity (Days)	Preoviposition Period (Days)	Oviposition Period (Days)	Postoviposition Period (Days)	Total Fecundity (Eggs/Female)	Egg Hatch (%)	Sex Ratio (% Females)
AD1	21.92 ± 2.77 b (n = 13)	24.40 ± 2.75 b (n = 10)	5.10 ± 0.35 a (n = 10)	8.30 ± 0.42 b (n = 10)	1.20 ± 0.25 a (n = 10)	25.20 ± 2.97 c (n = 10)	66.33 ± 2.91 a (n = 45)	45.00 ± 3.51 a (n = 26)
AD2	26.82 ± 1.60 a (n = 11)	30.15 ± 2.79 a (n = 13)	4.00 ± 0.30 b (n = 13)	9.46 ± 1.45 ab (n = 13)	2.08 ± 0.26 a (n = 13)	37.62 ± 1.65 b (n = 13)	67.33 ± 3.06 a (n = 45)	49.33 ± 5.24 a (n = 25)
AD3	26.08 ± 1.32 a (n = 13)	31.18 ± 3.12 a (n = 11)	4.82 ± 0.30 ab (n = 11)	10.09 ± 1.87 a (n = 11)	1.36 ± 0.24 a (n = 11)	42.09 ± 2.72 a (n = 11)	72.00 ± 4.82 a (n = 45)	48.00 ± 4.00 a (n = 30)

Means followed by a different letter in a column are significantly different (Tukey HSD test at *p* ≤ 0.05).

**Table 4:** Mean ± SE of the estimated life table parameters (n = 30) of *S. gilvifrons* fed on three different artificial diets.

Diet	Parameter					
	Intrinsic Rate of Increase (d^−1^)	Finite Rate of Increase (d^−1^)	Net Reproductive Rate (Offspring/Female)	Gross Reproductive Rate (Offspring/Female)	Mean Generation Time (d)	Doubling Time (d)
AD1	0.089 ± 0.013 b	1.093 ± 0.014 a	8.40 ± 2.36 b	14.76 ± 3.46 b	26.94 ± 2.59 a	8.20 ± 2.99 a
AD2	0.111 ± 0.011 ab	1.117 ± 0.012 a	15.43 ± 3.83 a	25.26 ± 4.29 a	24.70 ± 1.62 ab	6.41 ± 0.74 b
AD3	0.119 ± 0.013 a	1.26 ± 0.011 a	16.30 ± 3.52 a	32.38 ± 5.05 a	23.45 ± 1.56 b	5.92 ± 0.55 b

The bootstrap procedure was used to calculate the standard errors with 100,000 bootstraps. The means followed by different letters in each column are significantly different between diets using the bootstrap test.
